# Evaluation of a New Modification of Pancreaticogastrostomy after Pancreaticoduodenectomy: Anastomosis of the Pancreatic Duct to the Gastric Mucosa with Invagination of the Pancreatic Remnant End into the Posterior Gastric Wall for Patients with Cancer Head of Pancreas and Periampullary Carcinoma in terms of Postoperative Pancreatic Fistula Formation

**DOI:** 10.1155/2014/490386

**Published:** 2014-09-16

**Authors:** Mohamed Mazloum Osman, Walid Abd El Maksoud

**Affiliations:** General Surgery Department, Faculty of Medicine, University of Alexandria, Alexandria 21526, Egypt

## Abstract

*Background/Objectives*. Postoperative pancreatic fistula (POPF) remains the main problem after pancreaticoduodenectomy and determines to a large extent the final outcome. We describe a new modification of pancreaticogastrostomy which combines duct to mucosa anastomosis with suturing the pancreatic capsule to posterior gastric wall and then invaginating the pancreatic remnant into the posterior gastric wall. This study was designed to assess the results of this new modification of pancreaticogastrostomy. *Methods*. The newly modified pancreaticogastrostomy was applied to 37 consecutive patients after pancreaticoduodenectomy for periampullary cancer (64.86%) or cancer head of the pancreas (35.14%). Eighteen patients (48.65%) had a soft pancreatic remnant, 13 patients (35.14%) had firm pancreatic remnant, and 6 patients (16.22%) had intermediate texture of pancreatic remnant. Rate of mortality, early postoperative complications, and hospital stay were also reported. *Results*. Operative mortality was zero and morbidity was 29.73%. Only three patients (8.11%) developed pancreatic leaks; they were treated conservatively. Eight patients (16.1%) had delayed gastric emptying, one patient (2.70%) had minor hemorrhage, one patient (2.70%) had biliary leak, and four patients (10.81%) had superficial wound infection. *Conclusions*. The new modified pancreatogastrostomy seems safe and reliable with low rate of POPF. However, further prospective controlled trials are essential to support these results.

## 1. Introduction

Pancreaticoduodenectomy (PD) is a procedure commonly performed for malignant and some benign diseases of the pancreatic head [1]. In the past, mortality rate after pancreaticoduodenectomy was very high. Nowadays, with the advancement of imaging studies, surgical techniques, and perioperative management, the mortality rate of pancreaticoduodenectomy has decreased to 0–9%. However, in most of the recent series morbidity rate remains high (30–50%), even at high volume centers [2, 3].

Postoperative pancreatic fistula (POPF) is the most common complication of pancreaticoduodenectomy. It is a critical trigger of life-threatening complications such as intra-abdominal abscess and hemorrhage [4]. It leads to prolongation of hospital stay, severe morbidity, or even surgical mortality. The incidence of POPF is reported to be 0–17% based on a variety of definitions [5, 6].

In the literature, there is no consensus toward the superiority of either pancreaticojejunostomy (PJ) or pancreaticogastrostomy (PG) as the best method for reconstruction after pancreaticoduodenectomy. Most of the retrospective studies suggest an advantage of PG over PJ [7, 8]. On the other hand, recent meta-analysis of 10 RCTs showed no differences between PJ and PG techniques regarding the rates of postoperative complications, reoperation, and mortality [9]. Lack of uniform definitions of postoperative complications and different modifications of pancreatic reconstruction are considered limitations of the meta-analysis.

Being one of most important factors influencing the final outcome, the current focus in pancreatic resection surgery is directed toward attempts at reducing incidence of pancreatic fistula by constructing a safe pancreatic anastomosis. Several methods of anastomosing the pancreas to the stomach have been employed, including PG using several mattress sutures [10] and the so-called binding PG using two purse string sutures at the posterior gastric wall [11].

Here we report the results of a new technique for PG, which combines anastomosis between the pancreatic duct and the gastric mucosa with suturing the pancreatic capsule of the pancreatic remnants to the seromuscular layer of posterior gastric wall and invagination of the free end of pancreatic remnant into the posterior gastric wall using a purse string suture.

## 2. Patients and Methods

The calculated minimum sample size (*n*) for the present study was 24 patients based on Dahiru et al. [12], considering the highest reported incidence for POPF (0.17), with *Z*_*α*_ value =1.96 (for *α* = 0.05) and a maximum acceptable error of 0.15 (power = 85%), so the calculated minimum sample size (*n*) = (*Z*_*α*_^2^ × *P* × *Q*)/*D* ^2^, where *Z*_*α*_ is the *Z*-value for the selected level of confidence (1 − *α*) = 1.96; *P* is the estimated incidence in the population =17%, that is, 0.17; *Q* is the (1 − *P*) = 83%, that is, 0.83; and *D* is the maximum acceptable error = 0.15(1 − *β*). The study included all patients (37 patients) who were operated on by elective pancreaticoduodenectomy with pancreaticogastrostomy in Alexandria Main University Hospital, Alexandria, Egypt, between February 2009 and June 2013. Patients with operable periampullary carcinoma and cancer head of the pancreas were included in the study. Inoperable cases whether diagnosed preoperatively or intraoperatively were offered palliative stenting or bypass surgery and excluded from the study. 

*Preoperative Work-Up.* All patients were subjected to thorough history taking, clinical examination, and laboratory investigations including tumor markers CA 19.9 and CEA. Computed tomography scan was done for all patients. ERCP was done to selected cases when periampullary carcinoma was suspected to establish diagnosis. All patients signed an informed consent regarding their understanding of the procedure and its potential complications as well as their approval of participation in the research. 

*Operative Work-Up.* All patients were operated on by the same team of surgeons. 

**Figure 1:**
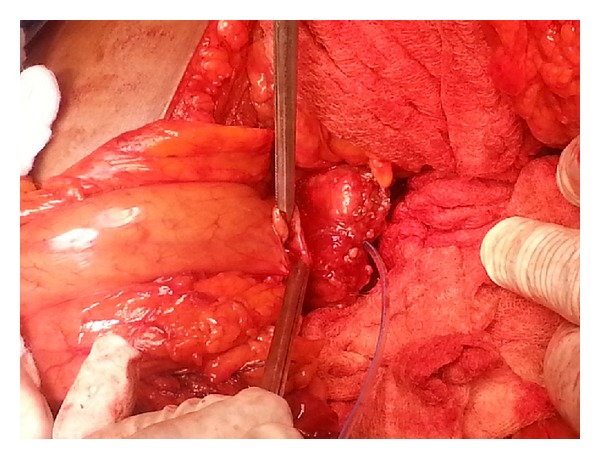
Pancreatic duct opposing the transverse full thickness opening in the posterior gastric wall.

**Figure 2:**
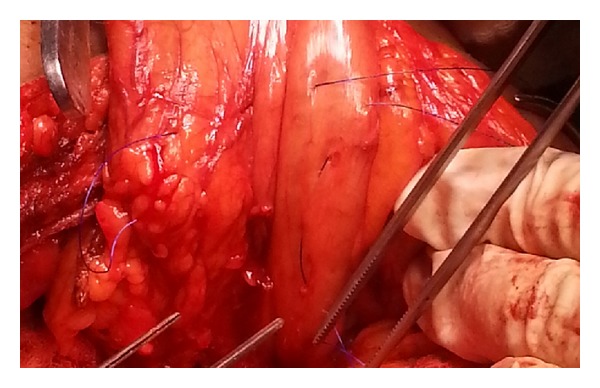
Purse string suture is made in the posterior gastric wall.

**Figure 3:**
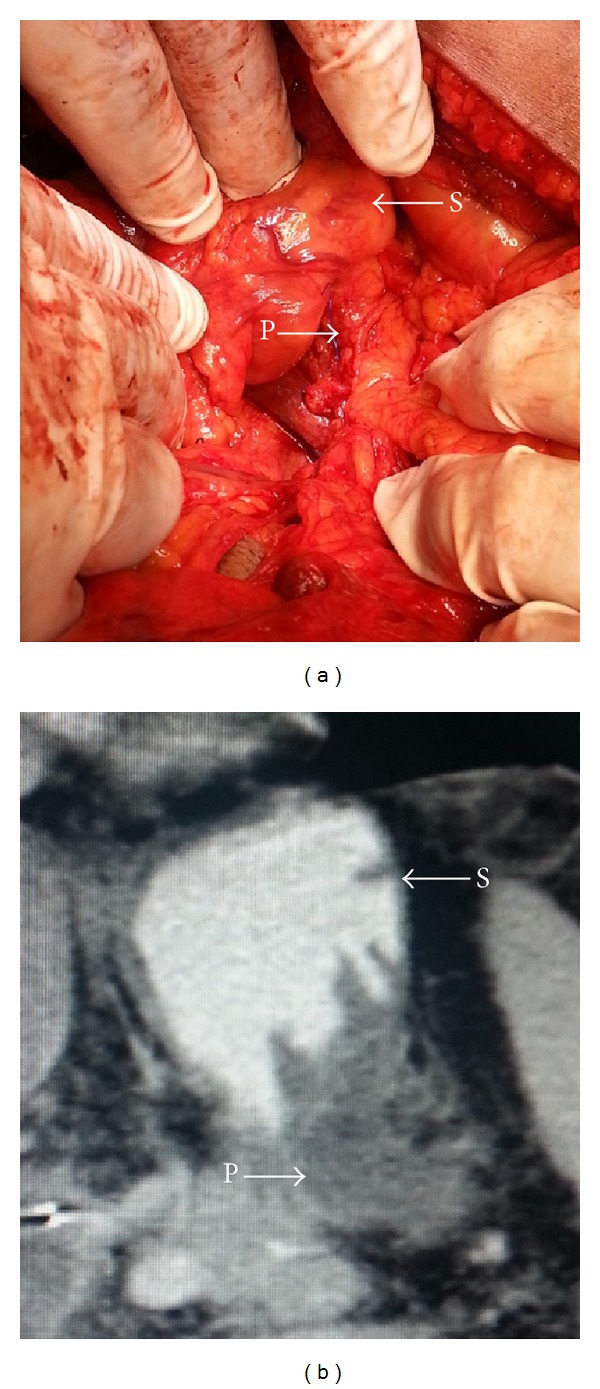
(a) Intraoperative invagination of the pancreatic remnant (P) into the posterior gastric wall (S); (b) postoperative CT scan showing invagination of the pancreatic remnant (P) into the posterior gastric wall (S).

**Figure 4:**
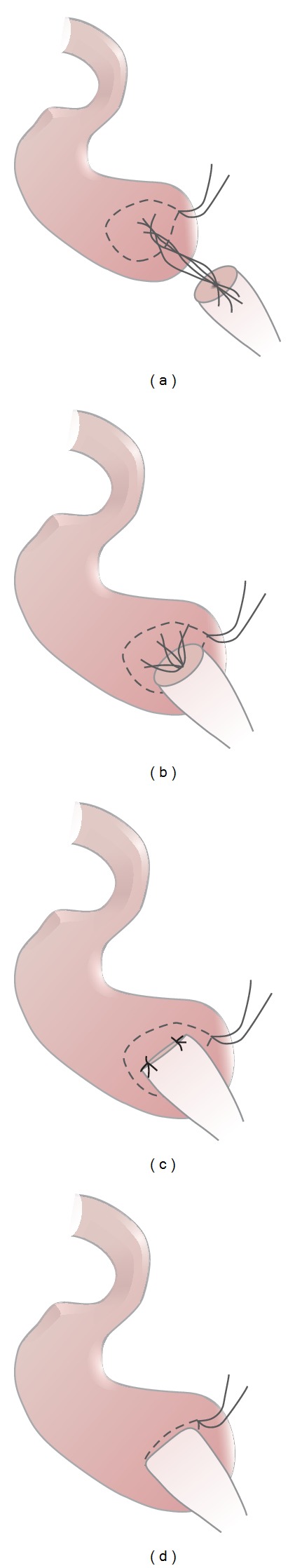
A diagram showing steps of the procedure. (a) A purse string is performed around a 5 mm stab incision in the posterior wall of stomach; then four sutures are performed between the pancreatic duct and gastric mucosa. (b) Tightening duct to mucosa sutures. (c) Sutures are taken between pancreatic capsule and seromuscular layer of the stomach. (d) Invagination of the pancreatic remnant into posterior wall of the stomach and tightening of the purse string suture.

*Operative Technique.* Technique of pancreatic division varies widely among surgeons, and there is no evidence that identifies a single method as superior. In our institute, the technique of pancreatic division either soft or fibrotic is the stapled technique using blue cartridge linear cutter stapler. Staple line length was 55 mm, number of staples rows were 4, open staple height was 3.85 mm, and closed staple height was 1.5 mm. Frozen section pathological examination of the resected specimens was routinely done to ensure free margin. Very few cases required further hemostasis as use of the linear cutter was very effective in controlling bleeding from the pancreatic edge. The pancreatic remnant was mobilized 2 to 3 cm from the splenic vein and the surrounding tissues. After removal of one or two staples from the stapled edge and identification of the pancreatic duct, a 10 or 12 Fr. polyethylene catheter passed into the main pancreatic duct to ensure its patency. The catheter was cut 1 cm from the pancreatic edge. Then a stab 5 mm transverse full thickness incision was made in the posterior wall of the stomach opposite to the pancreatic duct end (Figure 1). A purse string suture (Figure 2) was made in the posterior gastric wall around the opening in the posterior gastric wall using 2-0 polypropylene sutures, with a distance between the purse suture and the opening 1.5 times the distance between the pancreatic duct and the upper edge of the pancreas. The purse string suture was left loose. Next, anastomosis between the pancreatic duct and the gastric mucosa was done using four 4-0 polypropylene sutures at the four quarters. Keeping the catheter in the pancreatic duct with its edge passing into the stomach through the posterior gastric opening is essential during the duct to mucosa anastomosis to facilitate visualization of the duct and to prevent occlusion of the duct by sutures. It is kept in place at the end of the procedure and left to drop spontaneously and later pass naturally. Four 3-0 polyglactin sutures were secured between the capsule of the pancreas about 2 cm away from stapler line and about 1 cm deep in the pancreatic tissue and the corresponding area of seromuscular layer of the posterior wall of the stomach. These sutures were distributed at equal distances from each other. In cases of soft pancreas, the pancreatic sutures were taken a little bit deeper in the pancreatic parenchyma. Care was taken that the posterior suture must be done and kept loose before the anastomosis between the pancreatic duct and the gastric mucosa as the field will be blocked by the anastomosis and will make taking this suture very difficult and hazardous. Then, the posterior wall of the stomach is wrapped around the pancreatic remnant, while the purse string is tightened to ensure invagination of the pancreatic remnant (Figure 3). This maneuver was performed very gently to ensure tight wrapping of the posterior gastric wall around the pancreatic remnant without any tension on the anastomosis between the pancreatic duct and the gastric mucosa. Steps of the modified technique are shown in Figure 4. Furthermore, establishing of digestive tract continuity was obtained through end-to-side hepaticojejunostomy and side-to-side stapled gastrojejunostomy. All patients had two closed suction drains placed at the time of operation, one in close proximity to the pancreatic anastomosis and the other in the pelvis.

Operative time and intraoperative complications, if any, were reported. The texture of the pancreas whether soft, intermediate, or firm was also reported.

*Postoperative Work-Up.* All patients were admitted to ICU for the night of the operating day. Patients received Octreotide (100 *µ*g/8 h) until postoperative day 5. Proton pump inhibitor Omeprazole was given in a dosage of 40 mg/12 h for 7 days. The nasogastric tube was left in place until postoperative day 5 to protect the pancreaticogastrostomy. Volume and amylase activity of the fluid collected from drains were documented on postoperative days 1 and 5. The closed suction drains were removed on postoperative day 5, when amylase level of the drained fluid was no more than three times higher than the serum amylase. In cases when postoperative pancreatic fistula (POPF) occurred, the drains were left in situ until remission. POPF was classified according to IGSPF criteria [13] into type A fistulas without clinical impact, type B fistulas which needed maintenance of the drains longer than 3 weeks, and type C fistulas which needed clinical interventions like percutaneous drainage or reoperation. In case of POPF, quantity and quality of drained fluid were measured and documented daily and amylase level in the drainage fluid was tested upon surgeon's request.

Hospital stay, early postoperative complications, postoperative interventional aspiration, reoperation, and hospital mortality were reported in the postoperative period.

Univariate analysis was done to determine if there is a specific predictor affecting the occurrence of POPF.

### 2.1. Outcomes

The primary endpoint was to determine the rate of incidence of POPF following the new modification of pancreaticogastrostomy compared to what is mentioned in literature. POPF was diagnosed on the 5th postoperative day when amylase of the drained fluid was three times higher than the serum amylase.

Secondary endpoints included rate of mortality, early postoperative complications (delayed gastric emptying, hemorrhage, biliary leak, and wound infection), and hospital stay.

### 2.2. Statistical Analysis

The statistical analysis of data was done using excel program for figures and SPSS (SPSS, Inc., Chicago, IL, version 17). The description of data was done in the form of mean ± SD for quantitative data and frequency and proportion for qualitative data. The Chi-square test was applied for qualitative data and odds ratio for risk assessment. *P* value was considered as statistically significant if ≤0.05 at confidence interval 95%.

This trial is registered with ACTRN12614000481673.

## 3. Results

The study included a total of 37 consecutive patients who underwent PD with PG. There were 29 men (78.38%) and 8 women (21.62%), with a mean age of 60.39 ± 6.36 (range 42–71 years). BMI of the patients was 24.68 ± 4.16 Kg/M^2^ (range 15.64–30.91 Kg/M^2^). All patients underwent standard PD followed by the modified PG as described above. Twenty-four patients (64.86%) were suffering from periampullary cancer, while 13 patients (35.14%) suffered from cancer head of the pancreas. Thirteen patients (35.14%) had firm pancreatic tissue, 18 patients (48.65%) had soft pancreatic tissue, and 6 patients (16.22%) had intermediate texture of pancreatic tissue. Mean operative time was 221.5 ± 27.6 minutes (range 182–303 minutes). No intraoperative complications were encountered in the study.

The mean time of hospital stay was 12.9 ± 3.7 days (range 8–22 days). Regarding postoperative complications, there were no operative or postoperative deaths. Complications occurred in 11 patients (29.73%). Delayed gastric emptying (defined as intolerance of unrestricted oral diet after the tenth postoperative day) occurred in 8 patients (21.62%). Pancreatic leak occurred in 3 patients (8.11%). Two of them had intraoperative soft pancreas and one had an intermediate texture of the pancreatic remnant. Two were considered as type A leak and the other as type B according to IGSPF criteria [13]. One patient (2.70%) had minor biliary leak that was coming through the wound with no intra-abdominal collection. It was treated conservatively and stopped spontaneously after 4 days (biliary fistula was defined as persistence of biliary drainage for more than 5 days). One patient (2.70%) had bleeding from the pelvic drain. Bleeding was controlled after stopping heparin together with other conservative measures. Four patients (10.81%) had superficial wound infection. They were treated conservatively with wound care and antibiotics.

Although two of three patients with POPF were males and had soft pancreas, male sex and soft pancreatic texture were not statistically significant risk factors for POPF in univariate analysis. On the other hand, patients with POPF had significant longer hospital stay (Table 1).

## 4. Discussion

Reconstruction after pancreatoduodenectomy has become an issue of controversy during recent years [14]. Still the two common available options are PJ and PG. PG, first reported in 1946 by Waugh and Clagett [15], is preferred by many surgeons recently as many theoretical factors give the PG advantages compared to PJ. The proximity of the posterior gastric wall to the pancreatic remnant makes the anastomosis easy and with less tension. Nasogastric decompression of the stomach also helps to decrease the tension on anastomosis. In addition, the good blood supply of the stomach compared to small intestine leads to better healing. Furthermore, with PG, the pancreatic exocrine secretions enter the acidic gastric environment, where the low pH and lack of enterokinase prevent their activation. This lack of enzymatic activation may help to prevent autodigestion of the anastomosis. PG reduces the number of anastomoses in a single loop of retained jejunum and avoids creation of a long jejunal limb between the pancreatic and biliary anastomoses, where biliary and pancreatic secretions could collect and cause increased pressure, possibly resulting in tension at both the pancreatic and biliary anastomosis [16, 17]. In our institute, PG is the procedure used for reconstruction after pancreatoduodenectomy. Many authors support this practice. Yang et al. [9] showed a lower fistula rate (2.3%) after PG compared to (20.4%) after PJ group. Also relaparotomy rate of the PJ group was 52.9%. None of the patients who developed a pancreatic fistula following PG required relaparotomy because conservative measures succeeded in controlling the fistula. No mortality related to pancreatic fistula occurred in the PG group. Schlitt et al. [18], in a large series of patients, reported leak rates of 2.8% after PG and 12.6% after PJ. The mortality rate associated with leakage was 1.6% after PG and 5.2% after PJ. Pancreaticojejunostomy leaks were also associated with a high incidence of bile leaks at the hepaticojejunal anastomosis and took a longer time to close. On the other hand, Yeo et al. [19] in a prospective randomized trial found no differences between PG and PJ regarding incidence of complications including the pancreatic leak. However, it was a single center experience. We believe that further well designed multicentric studies with standardization of the definitions are required to clarify this issue.

POPF is the most troublesome complication of pancreatoduodenectomy, and various features that may influence the ultimate outcome of any pancreatic anastomosis have been critically studied [19]. Some preoperative and intraoperative predictive risk factors as well as technical factors were determined to be the most important factors influencing the incidence of POPF. Preoperative predictive factors such as old age (>70) and male sex showed significant risk for increasing the incidence of POPF in some studies [20, 21]. Matsusue et al. [21] found that preoperative high bilirubin level was associated with increase in morbidity and mortality. Pancreatic texture was found to be a very important intraoperative predictive risk factor. Patients with soft pancreas and friable pancreatic tissue have an association with high rates of POPF in many studies [22, 23]. Soft pancreas is usually seen with periampullary cancer rather than cancer head of pancreas. In this study, neither the age nor the high bilirubin level has statistical relation to incidence of POPF. On the other hand, male sex and soft pancreas were present in two of three patients who suffered from POPF in this study. However, this was not statistically significant. This may be attributed to the small number of patients with POPF in our study.

Technique of pancreaticoenteric anastomosis was subjected to critical studies in order to reduce incidence of POPF. Various technical modifications of the pancreaticogastric anastomosis such as duct to mucosa anastomosis and dunking technique were developed [10, 24]. However, there is still no clear evidence with or against any type of anastomosis [25]. Ohigashi et al. [10] reported a new modified technique describing the theoretical advantages of the binding and transfixing modifications. Bartsch et al. [24] reported another new technique for PG, which combines one binding purse string and two transfixing mattress sutures between the pancreatic stump and the posterior gastric wall. Zhu et al. [26] described pancreaticogastrostomy modification with double-binding continuous hemstitch sutures in the posterior gastric wall. In this study, we report a new modification for pancreaticogastric anastomosis. We combined duct to mucosa anastomosis with suturing the pancreatic capsule to posterior gastric wall and then invaginating the pancreatic remnant into the posterior gastric wall using a single purse string binding suture. Many studies [16] showed that mucosa to duct anastomosis has significant lower rates of POPF compared to invagination technique in PJ. We think that this may also be valid in PG. In addition, we agreed with Zhu et al. [26] that transfixing sutures through soft pancreas may carry risk of laceration and damage to pancreatic tissue. In soft pancreas, duct to mucosa anastomosis is safer. Some opinions have doubts regarding the possibility of making duct to mucosa anastomosis in soft pancreas in addition to difficulties in anastomosing the small duct diameter [16]. Nevertheless, we could cannulate the pancreatic duct using a narrow polyethylene catheter in all patients with soft pancreas (48.64% of patients included in the study) and once cannulation is performed, the anastomosis was performed with no technical problems. Sutures between the pancreatic capsule and posterior gastric wall are made in a gentle way just to occlude the space and prevent future collection that can increase pressure around the anastomosis. Invagination of the pancreatic remnant into the posterior gastric wall carries the theoretical advantages of preventing pressure on anastomosis and assisting in closure of the space between the pancreatic remnants and gastric wall. It also prevents collection of hematoma that may result from bleeding from the edge of the pancreatic remnant.

In conclusion, the new modified pancreaticogastrostomy by combining duct to mucosa anastomosis with suturing the pancreatic capsule to posterior gastric wall and then invaginating the pancreatic remnant into the posterior gastric wall using a single purse string binding suture showed promising results as regards POPF. It is safe and reliable. However, further prospective controlled trials with larger volume are essential to support these results.

## Figures and Tables

**Table 1:** Analysis for different risk factors affecting the occurrence of postoperative pancreatic fistula (POPF).

	Patients with POPF (*n* = 3)	Patients without POPF (*n* = 34)	*P* value
Age (years)			
Mean ± SD	61.50 ± 3.53	60.31 ± 6.54	0.750
Sex			
Male/female	2/1	27/7	0.529
BMI (Kg/Mr^2^)			
Mean ± SD	21.66 ± 2.90	24.89 ± 4.19	0.202
Diagnosis (number of patients)			
Periampullary cancer	1 (33.3%)	23 (67.6%)	0.270
Cancer head	2 (66.6%)	11 (32.4%)	
Preoperative bilirubin level (mg/dL)			
Mean ± SD	5.70 ± 1.27	5.09 ± 2.09	0.620
Pancreatic texture (number of patients)			
Soft	2 (66.6%)	16 (47%)	0.370
Intermediate	1 (33.3%)	5 (14.7%)	
Firm to hard	0 (0.0%)	13 (38.2%)	
OR time (minutes)			
Mean ± SD	209.50 ± 13.43	222.27 ± 28.53	0.450
Hospital stay (days)			
Mean ± SD	20.00 ± 2.82	12.41 ± 3.25	<0.001∗

*P < 0.05 is significant.
